# Complete mitochondrial DNA sequence of the alien hornet *Vespa velutina* (Insecta: Hymenoptera) invading Kyushu Island, Japan

**DOI:** 10.1080/23802359.2018.1437823

**Published:** 2018-02-09

**Authors:** Ryoichi Takahashi, Hisashi Okuyama, Yûsuke N. Minoshima, Jun-Ichi Takahashi

**Affiliations:** a Faculty of Life sciences, Kyoto Sangyo University, Kyoto, Japan;; b Kitakyushu Museum of Natural History and Human History, Kitakyushu, Japan

**Keywords:** Asian hornet, origin, genetic distance, alien species, Kyushu Island

## Abstract

We analyzed the complete mitochondrial genome of the invasive Asian hornet *Vespa velutina* from Kyushu Island, Japan. The mitochondrial genome of *V. velutina* was identified as a circular molecule of 16,388 bp. We predicted that the genome contains 13 protein-coding genes (PCGs), 22 tRNA genes, and 2 rRNA genes, along with one A + T-rich control region. The average AT content is 81.68%. Molecular phylogenetic analysis using the 13 mitochondrial PCGs from 11 closely related taxa of Vespidae indicated that the *V. velutina* invading the Japanese Islands of Kyushu and Tsushima have a common origin.

Naturalization of the invasive Asian hornet *Vespa velutina* has resulted in a general decline in native hornet populations and apiculture and an increase in sting injuries across non-native countries, including South Korea, Japan, and some European countries (Takahashi and Takahashi 2016; Martin 2017). In Japan, *V. velutina* was first observed on Tsushima Island in 2012 (Sakai and Takahashi 2014; Takahashi et al. 2015, 2016). In 2015, a *V. velutina* nest was found in Kitakyushu City on Kyushu Island in Japan (Minoshima et al. 2015). On the basis of partial mitochondrial DNA sequence analysis, Takeuchi et al. (2017) suggested that the *V. velutina* found in Kyushu Island had invaded the Island from either South Korea or Tsushima Island. To facilitate effective prevention of *V. velutina* introduction and establishment, genetic data are necessary to identify the invasive paths of this species. Here, we report the complete mitochondrial genome of the invasive hornet *V. velutina* found in Kitakyushu City, which will enhance our understanding of its invasion routes in Japan, and thus aid in its eradication.

Adult workers of *V. velutina* were collected at the time of destroying the nest found in Kitakyushu City (these specimens are stored in the Kitakyushu Museum of Natural History and Human History). Genomic DNA isolated from one worker was sequenced using an Illumina NextSeq 500 sequencer (Illumina Inc., USA). The resultant reads were assembled and annotated using the MITOS web server (Bernt et al. 2013), MEGA6 (Tamura et al. 2013), and GNETYX v.10 (Genetyx Corporation, Japan). Phylogenetic analysis was performed using the March 2011 version of TREEFINDER (Gangolf Jobb, Germany) based on nucleotide sequences of the 13 protein-coding genes (PCGs).

The *V. velutina* mitochondrial genome forms a 16,388-bp closed loop (accession number AP018483). It is representative of hornet mitochondrial genomes and is consistent with the genomic organization common in *V. velutina*, in that it comprises 13 PCGs, 22 tRNA genes, and 2 rRNA genes, as well as an A + T-rich control region. The average AT content of the *V. velutina* mitochondrial genome is 81.68%. Similar to other hornet mitochondrial genomes (Okuyama et al. 2017; Takahashi et al. 2017), the heavy strand was predicted to contain 9 PCGs and 14 tRNA genes, and the light strand was predicted to contain 4 PCGs, 8 tRNA, and 2 rRNA genes. The genes *ND4* and *ND4L* shared seven nucleotides. Of the 13 PCGs, the initiation codons ATC, ATG, ATT, and ATA were found in one, six, five, and one genes, respectively, whereas TAA is the termination codon in all these genes. Phylogenetic analysis using the 13 mitochondrial PCGs from 11 closely related taxa of Vespidae indicated a sister relationship between the *V. velutina* collected from Kyushu and Tsushima islands (Figure 1). The mitochondrial DNA sequences of *V. velutina* from these two Japanese islands matched completely, indicating that the invasive queens originated from the same maternal line. Complete sequence analysis of the *V. velutina* mitochondrial genome may provide important information regarding the origin and invasive routes of these alien hornets.

**Figure 1.**
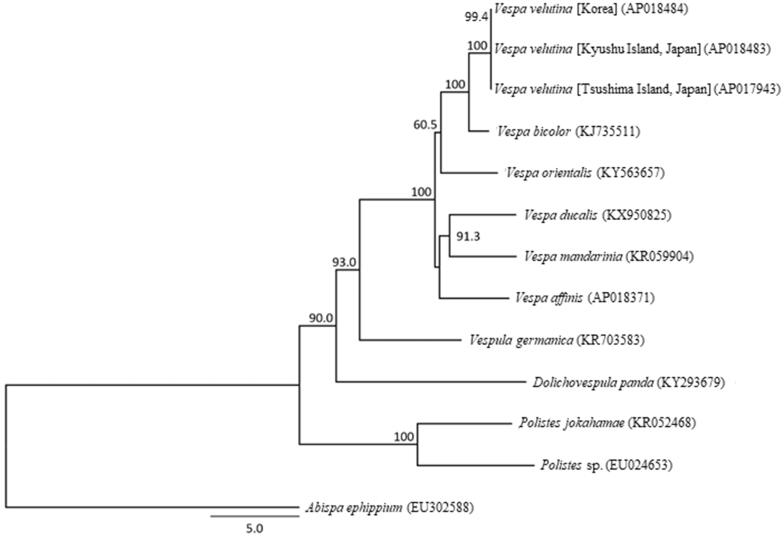
Phylogenetic relationships (maximum likelihood) of the Vespidae based on nucleotide sequences of the 13 protein-coding genes of the mitochondrial genome. The numbers at the nodes indicate bootstrap support inferred from 1000 bootstrap replicates. Alphanumeric terms indicate the GenBank accession numbers. *Vespa ducalis*, *V. orientalis*, *V. mandarinia*, *V. affinis*, *Vespula germanica*, *Dolichovespula panda, Polistes.* sp., *P. jakohamae,* and *Abispa ephippium* (Cameron et al. 2008; Chen et al. 2016; Song et al. 2016; Wei et al. 2016; Zhou et al. 2016; Fan et al. 2017; Haddad et al. 2017; Kim et al. 2017; Okuyama et al. 2017) were used as outgroup.
